# The Diagnostic Value of Quantitative CT Analysis of Ground-Glass Volume Percentage in Differentiating Epidermal Growth Factor Receptor Mutation and Subtypes in Lung Adenocarcinoma

**DOI:** 10.1155/2019/9643836

**Published:** 2019-03-06

**Authors:** Hexiang Wang, Hongwei Guo, Zeguo Wang, Bao Shan, Jizheng Lin

**Affiliations:** ^1^Department of Radiology, The Affiliated Hospital of Qingdao University, Qingdao, Shandong, China; ^2^Department of Surgery Center, The Qingdao Women and Children Hospital, Qingdao, Shandong, China; ^3^Department of Radiology, QingDao Municipai Hospital, QingDao, Shandong, China

## Abstract

**Objective:**

To retrospectively investigate computed tomographic (CT) quantitative analysis of ground-glass opacity (GGO) volume percentage and morphologic features of resected lung adenocarcinomas according to epidermal growth factor receptor (*EGFR*) mutation status and subtypes.

**Methods:**

Amplification refractory mutation system was used to detect mutations in the EGFR gene. Distribution of demographics and GGO volume percentage were performed according to EGFR mutation status and subtypes.

**Results:**

EGFR mutations were significantly more frequent in women (55.2% vs. 37.0%,* p*=0.001) and in never-smokers (59.5% vs. 38.4%, p < 0.001) than those without EGFR mutation. GGO volume percentage was significantly higher in tumors with EGFR mutation than in tumors without EGFR mutation (52.8±25.7% vs. 29.0±20.7%,* p* < 0.001). The GGO volume percentages in tumors with exon 21 mutation and EGFR mutation showed a significant difference compared with those without EGFR mutation (*p* < 0.001, area under the curve=0.871, sensitivity=94.6%, specificity=73.8%, and p < 0.001, area under the curve=0.783, sensitivity=69.9%, specificity=75.4%, resp.), with cut-off values of 37.7% and 34.3% in receiver operating characteristic curve analysis.

**Conclusion:**

GGO volume percentage in adenocarcinomas with EGFR mutation was significantly higher than that in tumors without EGFR mutation, and adenocarcinomas with exon 21 mutation showed significantly higher GGO volume percentage than in tumors with exon 19 mutation and those without EGFR mutation. Our results indicate that GGO volume percentage cut-off values of more than 37.7% and 34.3% were predictors of positive exon 21 mutation and EGFR mutation, respectively.

## 1. Introduction 

Epidermal growth factor receptor (EGFR) gene mutations are related to some specific characteristics, such as no smoking histology, female gender, adenocarcinoma, and Asian populations [1, 2]. Tumors with* EGFR *mutations are also closely associated with high sensitivity to treatment with EGFR tyrosine kinase inhibitors (TKIs) [2–4]. Some studies have shown that lung cancer patients with EGFR mutation who are treated with targeted TKIs, such as erlotinib, afatinib, or gefitinib, show longer progression-free survival and higher objective radiographic response rates than patients treated with standard first-line chemotherapy [4, 5]. However, lung cancer patients without EGFR mutation who receive gefitinib show shorter progression-free survival compared with patients treated with platinum-based chemotherapy [3]. The two most common types of* EGFR *mutation are exon 19 deletion and exon 21 missense mutation. However, whether these* EGFR *mutated cancers show differences in tumor characteristics is not clear [6, 7]. of great importance to patients with adenocarcinoma.

Computed tomography (CT) is the standard imaging modality for staging of lung cancer. Identification of correlations between CT features and lung adenocarcinoma subtypes associated with gene mutations is important and would help redefine recent existing staging and diagnostic paradigms and thus be of clinical benefit. Previous studies have attempted to identify specific CT features predictive of EGFR gene mutation. Most CT features showed no associations with EGFR status, except for ground-glass opacity (GGO) in tumors [8]. Several reports showed that GGO proportion was closely related with EGFR mutation status and was useful for stratifying EGFR mutation status [8, 9]. GGO proportion was also related to the lung adenocarcinoma histological subtype [8, 10, 11].

The purpose of this study was to retrospectively evaluate quantitative CT features of GGO volume percentage that correlate with* EGFR *mutation status and subtypes to assess the association and differences between tumors with EGFR mutations and those without EGFR mutation adenocarcinoma and in the tumoral mutations of EGFR subtypes.

## 2. Materials and Methods

### 2.1. Patients

A total of 309 consecutive patients were retrospectively analyzed (165 men and 144 women; mean age 51 years; range, 27–81 years). All the patients underwent curative surgical resection for lung adenocarcinoma with GGO between November 2014 and November 2017. EGFR status was available for all patients; a total of 163 adenocarcinomas had EGFR mutation, and 146 adenocarcinomas were without EGFR mutation.

### 2.2. CT Scanning

CT imaging was performed by using one of two CT systems (Discovery CT750 HD, GE Healthcare, Milwaukee, WI, USA; Somatom Sensation 64, Siemens, Erlangen, Germany; Somatom Definition Flash CT, Siemens). CT parameters were as follows: 120 kVp; 200 mAs; beam pitch, 1–5; slice thickness, 1 mm; slice gap, 1 mm; matrix, 512 ness. Each CT image covered the thoracic inlet to the level of adrenal gland.

### 2.3. Visual Analysis of Imaging Data

CT images were independently reviewed by two thoracic radiologists (both with more than 5 years of experience in chest CT interpretation) who independently interpreted CT images. Both radiologists were aware that patients had surgically resected lung adenocarcinomas but were blinded to the pathologic and EGFR test results. The radiologists first reviewed the morphologic characteristics, and the presence or absence of GGO with solid portion, air bronchogram, lobulated border, bubble-like lucency, notch, cavitation, and roundness was assessed. Bubble-like lucency was defined as small areas of air attenuation within the lesions. A lobulated border was defined when a surface part of a lesion showed a shallow wavy configuration, except for regions abutting the pleura [12, 13]. A notch was defined as V-shaped indentation of the border deeper than 3 mm [14]. A round tumor was defined for tumors with nearly identical maximum and perpendicular diameter without a notch. All tumors were categorized with pure solid nodules or GGO-containing nodules.

### 2.4. Quantitative Computer-Aided Volumetric Measurement of Imaging Data

**Figure 1:**
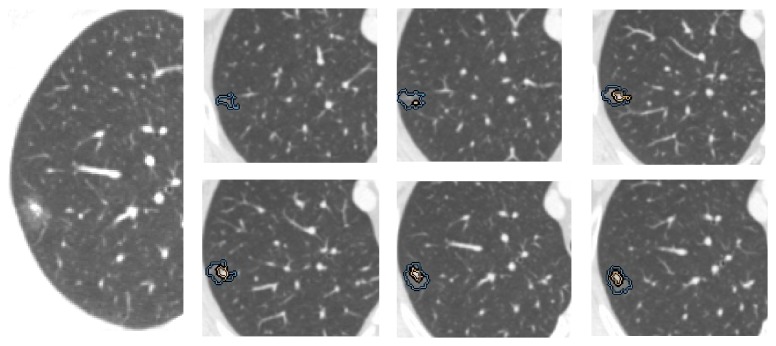
CT images use semiautomated segmentation and quantification of GGO volume percentage in a lepidic predominant invasive adenocarcinoma with EGFR mutation and GGO-containing nodule in a 65-year-old female. The tumor outer contour (blue line) and contour of the inner solid nodule (yellow line) are overlapped on each transverse image. The total tumor volume is 1.08 cm^3^ and the GGO volume percentage is 71.3%.

The GGO volume percentages were measured using a postprocessing workstation on Siemens Somatom Definition Flash CT. First, the entire tumor mass and the solid part were separated from surrounding anatomic structures using a semiautomated segmentation algorithm. Next, radiologists determined the specific boundary of the tumor lesion and the solid nodule by visual inspection. The computer then automatically calculated the volume of the entire mass and GGO volume percentage after the semiautomated segmentation and manual correction. Imaging for one of the patients is shown in Figure 1.

### 2.5. Histologic Evaluation and Molecular Analysis

All resected specimens were formalin fixed and stained with hematoxylin-eosin according to the standard procedure of our hospital. One board-certified pathologist (with 10 years of experience in pathologic diagnosis of lung cancer) examined the pathologic specimens and recorded the pathologic subtype. The mutation status of EGFR exons 18, 19, 20, and 21 was determined with the amplification refractory mutation system.

### 2.6. Statistical Analysis

Statistical analyses were performed using SPSS 22 (SPSS Inc., Chicago, IL, USA). GGO volume percentage and age are expressed as mean ± standard deviation and compared using independent t-tests or Mann-Whitney U-test, as appropriate. Clinical findings including sex, smoking status, and visual CT features were analyzed using *χ*2 test or Fisher's exact test, as appropriate. GGO volume percentage values were analyzed using receiver operating characteristic (ROC) curve analysis that was used to evaluate the diagnostic value of the GGO volume percentage values, including determination of an appropriate cut-off value. Statistical significance was determined when P was less than 0.01.

## 3. Results

### 3.1. Characteristics of Patients with Adenocarcinoma according to EGFR Mutation Status

The characteristics of the patients included in this study are summarized in Table 1. Among the 309 patients, a total of 163 (52.8%) adenocarcinomas showed EGFR mutation. EGFR mutations were significantly more frequent in women (55.2% vs. 37.0%,* p*=0.001) and in never-smokers (59.5% vs. 38.4%,* p* < 0.001) than those without EGFR mutation. No significant difference was detected between the age of patients with EGFR mutation (mean age, 51±13 years; range, 27–81 years) and those without EGFR mutation (mean age, 50±14 years; range, 27–81 years).

### 3.2. Imaging Characteristics according to EGFR Mutation Status

Imaging characteristics according to EGFR mutation status are summarized in Table 2. No significant differences were observed in the imaging features between the two groups according to EGFR mutation status, including the presence or absence of GGO, air bronchogram, bubble-like lucency, lobulated border, notch, cavitation, or roundness (all* p* > 0.05). Only GGO volume percentage showed a significant difference between the two patient groups. GGO volume percentage was significantly higher in tumors with EGFR mutation (52.8±25.7%) than in tumors without EGFR mutations (29.0±20.7%) (*p* < 0.001).

### 3.3. Clinical Characteristics and GGO Volume Percentage according to EGFR Mutation Subtype

EGFR mutation subtypes and correlations with sex, smoking status, and GGO volume percentage are summarized in Table 3. Among the EGFR mutations detected in the current patient group, exon 19 and exon 21 mutations accounted for the majority of EGFR mutation subtypes (92.0%). Exon 18 deletion or missense mutation was detected in 5 patients (3.1%), and exon 20 insertion was detected in 8 patients (4.9%). Tumors with exon 18, exon 19, or exon 20 mutations showed no differences in sex, smoking status, or GGO volume percentage compared with tumors without EGFR mutation (all* p* > 0.01). Exon 21 missense mutation was more frequent in never smokers (*p *** < **0.001) compared with EGFR without tumors. GGO volume percentage in tumors with exon 21 missense mutations was significantly higher than that in tumors with exon 19 deletion and tumors without EGFR mutation (*p *< 0.001 and* p *< 0.001, resp.).

### 3.4. ROC Analysis of GGO Volume Percentage according to EGFR Mutation Status

**Figure 2:**
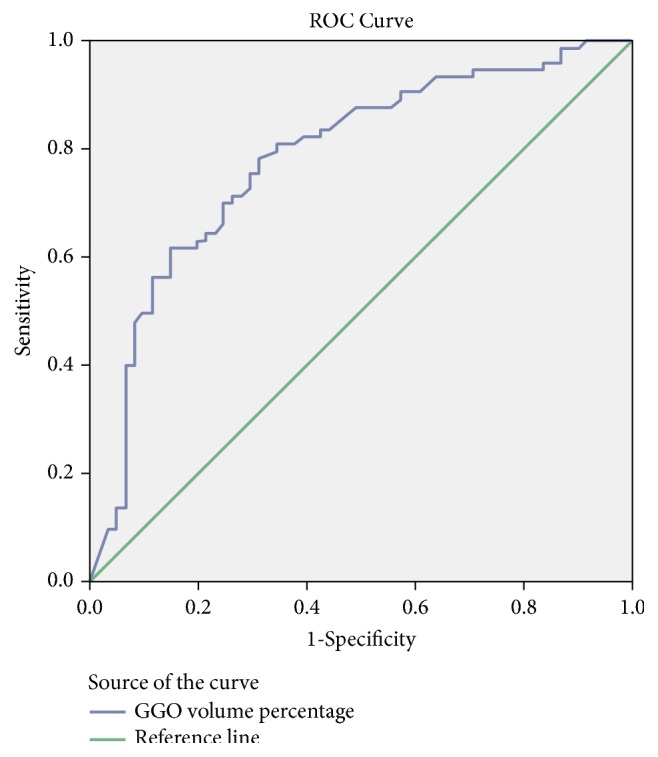
Receiver operating characteristic curve for GGO volume percentage values in adenocarcinomas with EGFR mutation compared with adenocarcinomas without EGFR mutation.

**Figure 3:**
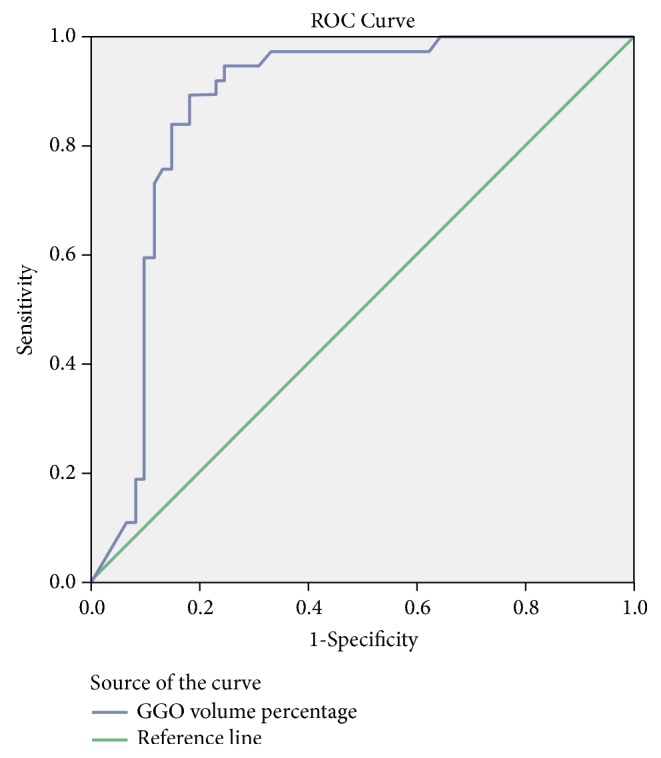
Receiver operating characteristic curve for GGO volume percentage values in adenocarcinomas with exon 21 missense mutation compared with adenocarcinomas without EGFR mutation.

ROC analysis revealed a significant difference in GGO volume percentage in adenocarcinomas with exon 21 missense and EGFR mutation compared with tumors without EGFR mutation (*p* < 0.001, area under the curve=0.871, sensitivity=94.6%, specificity=73.8%, and* p* < 0.001; area under the curve=0.783, sensitivity=69.9%, specificity=75.4%, resp.), with an estimated cut-off value of 37.7% and 34.3% in ROC analysis (Figures 2 and 3). The GGO volume percentage of exon 19 deletion displayed no significant difference in adenocarcinomas without EGFR mutation (*p*=0.059, area under the curve=0.627).

## 4. Discussion

Many technical methods are available for the detection of EGFR mutations; however, these methods are usually costly. In addition, due to the low percentage of tumor cells, the accurate detection of mutations is difficult and rebiopsy would likely be needed. In this study, we examined whether GGO volume percentage determination through routine CT may noninvasively differentiate adenocarcinomas with EGFR mutations from those without EGFR tumors, with the benefit of not incurring adding additional costs.

Our results showed that EGFR mutations were significantly more frequent in women and in never-smokers than in those without EGFR mutation, consistent with previous studies [8]. In addition, GGO volume percentages were significantly higher in patients with primary lung adenocarcinomas with EGFR mutation than in adenocarcinomas without EGFR mutation. Exon 19 or 21 mutations accounted for the majority of EGFR mutation subtypes (92.0%). GGO volume percentage in tumors with exon 21 missense mutation was significantly higher than that in tumors with exon 19 mutation and tumors without EGFR mutation.

The identification of EGFR mutations in lung adenocarcinoma according to response to EGFR-targeting TKIs have found the correlation between clinical findings and prognosis in patients with lung adenocarcinomas [15, 16]. Although some studies have identified an association of EGFR mutation with imaging features in lung adenocarcinomas, the results have been conflicting [7, 17–20]. One study reported no correlation between general CT features and EGFR mutation status [18]. Here we observed no significant difference in morphology imaging features according to EGFR mutation status, which is mostly consistent with the previous report. In contrast, another study reported that GGO was more frequently detected in EGFR-mutated tumors than in EGFR wild-type tumors [19].

There were no significant differences found in imaging features according to the past study, that no characteristic CT feature suggests EGFR mutation status [18]. However, we attempted to detect specific CT features predictive of EGFR mutation. We found that the GGO volume percentage was significantly higher in patients with primary lung adenocarcinomas with EGFR mutation compared with those without EGFR mutation. This finding is consistent with past reports that showed a close relationship between adenocarcinoma subtype and lesion density at CT [10, 11]. Lederman et al. [11] reported a strong correlation between GGO and lepidic subtype and between CT solidity and solid subtype. To the best of our knowledge, few reports have examined the GGO volume percentage values in adenocarcinomas with EGFR mutation compared with adenocarcinomas without EGFR mutation. In this study, ROC curves comparing the diagnostic performance of the GGO volume percentage values showed an area under the curve value of 0.783, which demonstrated good validity for the diagnosis of adenocarcinomas with EGFR mutation. The optimal cut-off point for differentiating adenocarcinomas with EGFR mutation from adenocarcinomas without EGFR mutation was 34.3%, with values above this threshold being likely to represent adenocarcinomas with EGFR mutation. This finding showed that GGO volume percentage was closely related with EGFR mutation status and was useful for stratifying EGFR mutation status [8].

Little is known about the correlations between EGFR mutation subtypes and imaging features. Several studies have examined the importance of EGFR mutation subtype in patient prognosis. Choi et al. [21] reported longer progression-free survival for patients with advanced adenocarcinoma with exon 19 deletion of EGFR who were treated with EGFR TKI therapy. Lee et al. [7] found that exon 21 mutation was more frequent in lepidic predominant adenocarcinomas and that the GGO volume percentage in tumors with exon 21 mutation is significantly higher than that in EGFR wild-type tumors. In our study, exon 21 mutation was related to a higher GGO volume percentage than tumors with exon 19 deletion and tumors without EGFR mutation status, which is consistent with the study by Lee et al. [7].

ROC curves evaluating the diagnostic performance of the GGO volume percentage values showed the area under the curve as 0.871, demonstrating good validity for the diagnosis of adenocarcinomas with exon 21 missense mutation. The optimal cut-off point for differentiating adenocarcinomas with exon 21 missense mutations from adenocarcinomas without EGFR mutation was 37.7%, with values above this threshold being likely to represent adenocarcinomas with exon 21 missense mutations. Growing evidence has suggested the importance of GGO volume percentage in the prognosis of lung adenocarcinomas [17, 22–24], and our results support the proposal that the radiogenomic relation to lung adenocarcinomas can be a useful method for predicting diagnosing results.

Sun et al. previously reported that no significant difference was detected between GGO volume percentage in tumors with exon 21 missense mutation and in tumors with exon 19 missense mutation in EGFR [8]. Our results are different from these findings. One possible explanation might be the small sample size of tumors with exon 19 and exon 21 mutations in both studies, and there may have been inherent selection bias in our study population.

This study had several limitations. First, this study was a retrospective review in a single institution in East Asia, and there may have been inherent selection bias in the research population. Second, the number of samples was not large enough to obtain a sufficient conclusion. Third, the interobserver, in observing CT images, variability was not evaluated.

In conclusion, our results showed that GGO volume percentage in adenocarcinoma with EGFR mutation was significantly higher than that in adenocarcinoma without EGFR mutation. Adenocarcinoma with exon 21 mutation showed a significantly higher GGO volume percentage than in tumors with exon 19 mutation and those without EGFR mutation. GGO volume percentages on CT images of more than 37.7% and 34.3% were predictors of positive EGFR exon 21 mutation and EGFR mutation, respectively. The GGO volume percentage on CT was a predictor of EGFR and exon 21 mutation in lung adenocarcinoma.

## Figures and Tables

**Table 1:** Patient Characteristics.

Characteristic	EGFR Mutation+	EGFR Mutation-	P Value
No. of patients	163	146	
Age (y)*∗*	51 ± 13	50 ± 14	p = 0.854
Sex			
Female	90	54	p = 0.001
Male	73	92	
Smoking			
Never smoked	97	56	p < 0.001
Smoker	66	90	

Note: Age compared using independent t-tests.

The rest groups were analyzed using *χ*2 tests.

**Table 2:** CT Characteristics.

CT Feature	EGFR Mutation+	EGFR Mutation-	P Value
No. of patients	163	146	
GGO volume percentage (%)	52.8 ± 25.7%	29.0 ± 20.7%	P < 0.001
Morphologic CT feature			
Air bronchogram	35	29	p = 0.727
Bubblelike lucency	21	16	p = 0.603
Cavity	19	25	p = 0.170
Notch	65	43	p = 0.550
GGO with solid portion	73	61	p = 0.594
Lobulated border	59	48	p = 0.540
Round	21	13	p = 0.264

Note: Age compared using independent t-tests.

The rest groups were analyzed using *χ*2 tests.

**Table 3:** Sex, smoking history, and GGO volume percentage according to EGFR mutation subtype.

Subtype	N	Sex			Smoking history			GGO	
		Women	Men	p-value	Never-smoker	Smoker	p-value	volume percentage (%) (range)	p-value
Exon 19 deletion	76	39	37	p = 0.040^||^	43	33	p = 0.009^||^	40.8 ± 28.9%	0.064*∗*
Exon 21 missense	74	44	30	p = 0.002^||^	47	27	P < 0.001^||^	65.2 ± 19.3%	P < 0.001*∗*
Exon 20 insertion	8	4	4	p = 0.461^‡^	4	4	p = 0.331^‡^	38.1 ± 9.4%	0.039^§^
Exon 18 insertion	5	3	2	p = 0.298^‡^	3	2	p = 0.512^‡^	47.3 ± 5.7%	0.083^§^
EGFR-	146	54	92		56	90		29.0 ± 20.7%	
Total	309	180	129		163	146			* *

Note: p-value based on a comparison between adenocarcinomas with each EGFR mutation subtype and adenocarcinomas without EGFR mutation.

Significant differences were found in GGO volume percentage between adenocarcinomas with exon 19 deletion and adenocarcinomas with exon 21 missense mutations (p<0.001) using independent t-tests.

*∗* independent t-tests.

|| *χ*2 tests

‡ Fisher's exact test

§ Mann-Whitney U -test

## Data Availability

The data used to support the findings of this study are available from the corresponding author upon request.
